# Mapping rust resistance in European winter wheat: many QTLs for yellow rust resistance, but only a few well characterized genes for stem rust resistance

**DOI:** 10.1007/s00122-024-04731-9

**Published:** 2024-09-05

**Authors:** Thomas Miedaner, Wera Eckhoff, Kerstin Flath, Anne-Kristin Schmitt, Philipp Schulz, Johannes Schacht, Philipp Boeven, Wessam Akel, Hubert Kempf, Paul Gruner

**Affiliations:** 1 State Plant Breeding Institute, University of Hohenheim, 70599 Stuttgart, Germany; 2 Present Address: Kleinwanzlebener Saatzucht (KWS) KWS SAAT SE & Co. KGaA, Einbeck, Germany; 3 Institut für Pflanzenschutz in Ackerbau und Grünland, Julius Kühn-Institut (JKI), Stahnsdorfer Damm 81, 14532 Kleinmachnow, Germany; 4 Limagrain GmbH, 31226 Peine-Rosenthal, Germany; 5 Strube Research GmbH & Co. KG, Hauptstraße 1, 38387 Söllingen, Germany; 6 SECOBRA Saatzucht GmbH, Feldkirchen 3, 85368 Moosburg an der Isar, Germany; 7 Present Address: Sativa Rheinau, Chorbstr. 43, 8462 Rheinau, Switzerland

## Abstract

**Key message:**

Stem rust resistance was mainly based on a few, already known resistance genes; for yellow rust resistance there was a combination of designated genes and minor QTLs.

**Abstract:**

Yellow rust (YR) caused by *Puccinia striiformis* f. sp. *tritici* (*Pst*) and stem rust (SR) caused by *Puccinia graminis* f. sp. *tritici* (*Pgt*) are among the most damaging wheat diseases. Although, yellow rust has occurred regularly in Europe since the advent of the Warrior race in 2011, damaging stem rust epidemics are still unusual. We analyzed the resistance of seven segregating populations at the adult growth stage with the parents being selected for YR and SR resistances across three to six environments (location–year combinations) following inoculation with defined *Pst* and *Pgt* races. In total, 600 progenies were phenotyped and 563 were genotyped with a 25k SNP array. For SR resistance, three major resistance genes (*Sr24*, *Sr31*, *Sr38/Yr17*) were detected in different combinations. Additional QTLs provided much smaller effects except for a gene on chromosome 4B that explained much of the genetic variance. For YR resistance, ten loci with highly varying percentages of explained genetic variance (pG, 6–99%) were mapped. Our results imply that introgression of new SR resistances will be necessary for breeding future rust resistant cultivars, whereas YR resistance can be achieved by genomic selection of many of the detected QTLs.

**Supplementary Information:**

The online version contains supplementary material available at 10.1007/s00122-024-04731-9.

## Introduction

Yellow rust (YR) caused by *Puccinia striiformis* f. sp. *tritici* (*Pst*) and stem rust (SR) caused by *Puccinia graminis* f. sp. *tritici* (*Pgt*) are among the most damaging wheat diseases in Germany and on a global scale, respectively (Serfling et al. 2016). Their control is by fungicides and genetic resistances. No active fungicide ingredient is currently approved for control of SR in Germany. The availability of fungicides, especially the widely used azoles approved for control of YR, is more and more restricted by the European Union (Jess et al. 2014). Following the European guidelines for integrated pest management, chemical control is considered an inferior choice and preventive measures such as resistance should be preferred (DIRECTIVE 2009/128/EC). Consequently, resistance breeding must be intensified to maintain effective disease management for European wheat production.

Until 2011 *Pst* populations in Europe were mostly endemic with clonal propagation and slow adaptation; however, the situation changed with the appearence of the Warrior race (Hovmøller et al. 2016). Due to the broad virulence combination of this race,  some formerly resistant varieties became susceptible. In contrast to the earlier European races, the Warrior race had a clear upwards shift in temperature optimum for infection and thus could become established under warmer conditions, exemplified by its occurrence in Italy and Spain (Miedaner and Juroszek 2021). These events created a strong motivation for breeding programs to shift to YR resistance based on adult-plant resistances reputed for durable protection. For SR, local stem rust outbreaks across Europe occurred in Central Germany in 2013 after more than 50 years (Olivera Firpo et al. 2017). This put breeding for stem rust resistance back in focus (Flath et al. 2014; Saunders et al. 2019). In 2016, there was a severe stem rust outbreak in durum and bread wheat in Sicily (Bhattacharya 2017) and in the following year stem rust occurred on late-maturing wheat and barley in Central Sweden (Berlin 2017). Saunders et al. (2019) predicted a re-occurrence of stem rust as common disease in Western Europe. Recently, even in Ireland with its maritime climate, stem rust was detected in experimental plots at several locations (Tsushima et al. 2022).

The irregular occurrence of *Pgt* compared to *Pst* is likely due to two factors: a higher optimal infection temperature for *Pgt* and the dominance of early-maturing winter wheat varieties, that shorten the window for *Pgt* infection coming from the alternate host (*Berberis* spp.), whereas the source of *Pst* is overwintering infection of cereals (Flath et al. 2018). However, this might fundamentally change due to predicted higher temperatures in future (Miedaner and Juroszek 2021). Flath et al. (2014) showed that only *Sr31* remained effective against most isolates collected during the stem rust outbreak in Germany 2013. *Sr31* was introduced into wheat from “Petkus” rye in the 1930s (Schlegel and Korzun 1997) and was one of the most frequently used resistance genes used worldwide (Pathan and Park 2007; Olivera Firpo et al. 2017). In 1999, *Sr31* became ineffective due to a new race first identified in Uganda. The race called *Ug99* (or TTKSK) was virulent for *Sr31* and to some other widely used *Sr* genes (Singh et al. 2015). It or its descendants subsequently spread to all countries in eastern Africa and were also found in Yemen, Iran, and in 2023, Nepal (Patpour et al. 2024). *Sr31* remains effective in Central Europe (Flath et al. 2018; Zelba et al. 2022). Additionally, stem rust resistance of cultivars with *Sr38* ranged from fully resistant to moderately susceptible, while *Sr24* was fully effective in field experiments with German winter wheat cultivars (Flath et al. 2018).

Rust resistance is often inherited by monogenic, race specific all-stage resistance (ASR; synonym seedling resistance) that usually proves to have low durability (McIntosh et al. 1995; Chen 2005). Alternatively, resistance is expressed only at the post-seedling or adult plant stages (APR). APR is often conferred quantitatively by multiple genes or QTL conferring only partial resistance (Ellis et al. 2014; Miedaner 2016). More than 300 regions in the wheat genome have been associated with YR resistance (Bouvet et al. 2022a). Kumar et al. (2023) condensed 505 YR QTLs from 101 linkage-based interval mapping studies to 67 meta-QTLs (MQTLs) and further refined them to 29 high-confidence MQTLs. This result was confirmed by Pal et al. (2022) who claimed 368 QTLs for leaf rust resistance, 291 QTLs for SR resistance, and 487 QTLs for YR resistance from 152 studies. Among the QTL plethora 28 MQTLs provided resistance to all three rusts, each of the other 43 MQTLs provided resistance to combinations of two rusts. Similar results were recently reported by Tong et al. (2024).

More than 80 resistance genes for YR and 56 genes for SR have been permanently assigned (McIntosh et al. 2013, 2014, 2020) and several more, temporarily named. The majority of those genes confer race-specific ASR and have, or had, limited durability (McDonald and Linde 2002). Pyramiding major R-genes with partial APR has increased the durability of ASR-genes, whereas combinations of only ASR genes were questioned in regard to durability (Mundt 2018). A recent survey of SR resistance found three ASR genes in German and Czech winter wheat panels, namely *Sr24*, *Sr31*, and *Sr38/Yr17/Lr37* (Flath et al. 2018; Zelba et al. 2022). Some cultivars without ASR genes still showed substantial resistance in the field suggesting the presence of quantitatively inherited APR in German breeding material.

The objectives of this study were to: (1) identify already known and unknown APR genes/QTL for yellow rust and stem rust resistance in the available mapping populations of European elite winter wheat, and (2) to investigate whether there are genes that confer resistance to both YR and SR. Seven mapping populations with a total of 600 progenies were phenotyped across several environments artificially inoculated with *Pst* and *Pgt*.

## Materials and methods

### Plant material and pathogen isolates

Trials were conducted in cooperation with the Institute of Plant Protection in Field Crops and Grassland, Julius Kühn-Institut (JKI) in Kleinmachnow, LIMAGRAIN GmbH, Strube Research GmbH and Co. KG and Secobra Saatzucht GmbH. Mapping populations were constructed and provided by the three breeding companies (Table 1).

**Table 1:** Overview of the seven biparental populations and their phenotyping

Population	Type^a^	Breeding company	Cross	No. of progeny	No. of env. SR/YR	*N*_G_ marker^b^	*N*_G_ unique^c^
Pop1	RIL	Secobra	Axioma × Memory	94	3/6	91	91
Pop2	DH	Limagrain	Mocca × LG Stamm 1	86	3/4	91	79
Pop3	DH	Limagrain	Mocca × LG Character	91	3/4	91	86
Pop4	DH	Limagrain	Mocca × LG Stamm 2	92	3/4	91	85
Pop5	DH	Strube	Gedser × Memory	68	3/3	84	65
Pop6	DH	Strube	Spontan × Bonanza	97	3/3	97	92
Pop7	DH	Strube	Edward × KWS Montana	72	3/3	72	65

env., environment; SR, stem rust; YR, yellow rust; N_G_, number of genotypes

^a^RIL, recombinant inbred line; DH, doubled haploid line

^b^Number of genotyped progeny

^c^Number of genotyped progeny after removing genotypes with heterozygosity > 0.1 and correlation > 0.99

### Mapping populations

This study was conducted mainly for mapping of SR and YR resistance genes in commercially grown German elite winter wheat material. Parents were chosen after conducting association studies on two German winter wheat diversity panels. The first panel consisting of 79 varieties was screened for stem rust resistance at the seedling and adult plant stages (Flath et al. 2018). The second panel of 270 varieties was screened for yellow rust and stem rust resistance at the adult-plant stage (Miedaner et al. 2020). Seven populations (Pop1–Pop7) were developed from biparental crosses. Their parents had different seedling responses to SR and/or YR (Table 2). In total, there were 11 parents including two unreleased lines and nine registered varieties; some populations shared one parent (Table 1). Doubled-haploid (DH) lines were produced from F_1_ plants. Population 1 (Pop1) was a recombinant inbred line (RIL) population derived from the F_4_ individuals. Pop2 to Pop4 had same susceptible parent (Mocca). Pop5 to Pop7 were tested only in the second year. Each population had 68–97 genotypes (entries) tested in field trials with 72–97 entries per population genotyped. However, based on marker data some genotypes from the respective populations appeared to be identical (correlation based on marker data ≥ 0.99) and each group of identical genotypes was considered as one genotype. The high number of identical genotypes was probably caused by the DH production procedure. During regeneration, calli induced from anthers (microspores) can break into multiple pieces that give rise to genetically identical genotypes. Parents were always tested together with their respective progeny.

**Table 2:** Reaction of the parents for stem rust (SR) and yellow rust (YR) in the field (across all environments) and the seedling test with the same isolate used for field inoculation (characterization see Table S2); in the field a quantitative scale for disease severity, in the seedling test a qualitative scale was used; for the virulences of the races please refer to Table S2

Pop	Parent	SR		YR		Race	
		Field^a^	Seedling^b^	Field^a^	Seedling^b^	SR	YR
Pop1	Axioma	10.9	S	2.8	MR	HFCLB (2020)	Warrior
	Memory	1.0	R	21.4	R		
Pop1	Axioma	10.9	S	2.8	MR	TKTTF (2021)	Warrior
	Memory	1.0	R	21.4	R		
Pop2	Mocca	9.7	S	13.2	S	HFCLB	Benchmark
	Stamm 1	3.6	MR	0.6	R		
Pop3	Mocca	8.4	S	13.7	S	HFCLB	Benchmark
	LG Character	2.4	MR	2.9	MR		
Pop4	Mocca	9.3	S	14.0	S	HFCLB	Benchmark
	Stamm2	1.2	MR	8.8	MR		
Pop5	Gedser	36.7	S	3.6	S	TKTTF	Warrior
	Memory	1.4	R	1.0	R		
Pop6	Bonanza	8.4	S	2.3	R	TKTTF	Warrior
	Spontan	9.2	MR	1.1	R^c^		
Pop7	Edward	23.7	S	15.4	S	TKTTF	Warrior
	KWS Montana	4.7	S^c^	0.0	R		

^a^Quantitative scale of disease severity: 0–100%

^b^S, susceptible; MR, moderately resistant; R, resistant

^c^No isolate of the collection differentiated these parents

### Experimental design

The materials were tested in multi-environment trials (MET) at five locations, namely Berlin-Dahlem in Eastern Germany (DAL, 52.44° N, 13.27° E, 45 m above sea level [a.s.l.]), Stuttgart-Hohenheim in Southern Germany (HOH, 48.80° N, 9.20° E; 401 m a.s.l.), Lemgo in Western Germany (LEM, 52.2° N, 8.55° E, 100 m a.s.l.), Rosenthal-Peine (ROS, 52.18° N, 10.10° E; 73 m a.s.l.) and Söllingen in Northern Germany (SOL, 52.06° N, 10.55° E, 90 m a.s.l.) in two seasons, namely 2020 and 2021. Because the segregating populations were breeding material and proprietary to the respective breeding companies, the complete set of genotypes was only grown at locations managed by the University of Hohenheim (HOH) and the Julius-Kühn Institute (DAL). Only material of the respective proprietary populations was grown at breeding stations LEM, ROS, and SOL (Table S1). The material of each breeding company (Limagrain, Secobra, Strube) was randomized in separate trials using a resolvable incomplete block design with two complete replicates. Only Pop2 to Pop4 were tested at locations HOH and ROS in 2020 using a single complete replicate across locations (p-rep design) due to shortage of seed. Each entry was grown in two-row plots of 1–1.2 m length and 0.4 m width and sown with 40–60 kernels per row. In DAL, the materials were grown in four-row 0.5-m plots.

### Pathogen isolates and seedling tests

Segregating populations were inoculated with specific races that distinguished the respective parental lines based on a previous seedling test conducted by Institute of Plant Protection in Field Crops and Grassland, Julius Kühn-Institut (JKI) in Kleinmachnow (Tables 2 and S2). Not all pairs of parents showed a significant difference in a specific seedling test. *Pst* and *Pgt* inoculum for all populations and environments was produced by JKI as described in Hovmøller et al. (2017) and Olivera et al. (2015). The *Pgt* isolates used were identified as races TKTTF and HFCLB (nomenclature according to Roelfs and Martens 1988, actualized by FAO 2024) and the *Pst* isolates were identified as the Warrior (*PstS7*) and Warrior (–) Benchmark (*PstS10*) races (Table S2) (nomenclature according to GRRC 2023a; Hovmøller et al. 2022).

Seedling tests were performed as previously described by Jin et al. (2007), Olivera et al. (2015), and Hovmøller et al. (2017). In short, fully expanded primary and secondary leaves of six to ten seedlings per line were inoculated 10 days after planting. All assessments were repeated in separate experiments. Seedling infection types were determined 18 days after inoculation for *Pst* following a 0–9 scale (Hovmøller et al. 2017): 0 = no visible disease symptoms (immune), 1 = minor chlorotic and necrotic flecks, 2 = chlorotic and necrotic flecks without sporulation, 3–4 = chlorotic and necrotic areas with limited sporulation, 5–6 = chlorotic and necrotic areas with moderate sporulation, 7 = abundant sporulation with moderate chlorosis, 8–9 = abundant and dense sporulation without notable chlorosis and necrosis. Infection types 7 to 9 were categorized as susceptible, 4 to 6 as moderately resistant, and 0 to 3 as resistant. For *Pgt* a 1–6 scale was used: 1 = no visible disease symptoms (immune), 2 = hypersensitive flecks, 3 = small uredinia with hypersensitive reactions, 4 = small to medium-sized uredinia surrounded by chlorosis, 5 = medium-sized uredinia with/without chlorosis, 6 = large uredinia without chlorosis. Infection types 5 to 6 were categorized as susceptible, 3 to 4 as moderately resistant, and 1 to 2 as resistant.

### Inoculation and data collection

Field trials were artificially inoculated twice with *Pst* at two-week intervals at early tillering (plant stage BBCH 21–23). *Pgt* was also inoculated twice at the end of heading and mid-flowering (BBCH 59–65). Urediniospores (100 mg per 100 m^2^) of *Pst* and *Pgt* spores were suspended in a 0.1% agar and applied by a microsprayer (Micron Ulva, Bromyard, Herefordshire, UK) across the entire plot area.

Rust response data were collected by visual scoring at several scoring dates to ensure at least one optimum date with maximum trait differentiation. Scoring was as the percentage area of infection per plot; leaf area for YR, and for SR, the area second uppermost leaf (Flag-1) and the node. Scoring of YR started when most genotypes showed mild symptoms. SR was scored at BBCH 87–89, when uredinia and telia were well developed. The number of scoring dates varied between trials and ranged from one to four for YR and from one to three for SR. Disease development differed between environments. In some environments SR reactions were not recorded due to low infection (Table S1).

### Phenotypic data analyses

Phenotypic analysis was conducted in a two-stage approach. The statistical models were computed using asreml-R ver. 4.1.0.160 (Butler 2021).

#### First stage

Best linear unbiased estimators (BLUEs) with standard errors (SEs) were calculated for each trait assessed in the *t*th environment *l* based on the following mixed model:

1 $$y_{tijklm} = \mu_{t} + g_{tij} + r_{tk} + b_{tkl} + e_{tijklm}$$

The plot value is modeled by the general intercept μ, the *i*th genotype *g*, the *k*th replicate *r*, the *l*th block* b* and the residual error *e*. To estimate BLUEs, *g* was modeled as fixed effect and all other effects as random. To assess repeatability (location-wise entry-mean heritability) by $Rep=\frac{{\sigma }_{G}^{2}}{{\sigma }_{G}^{2}+\frac{\overline{v}}{2} }$ the mean variance of a difference $\overline{v }$ was calculated and in a separate model the genotype was fitted as random to estimate genetic variance ${\sigma }_{G}^{2}$ (Piepho and Möhring 2007). In case of SR and YR, the same model was run for each assessment, but in the final analysis only the mean (or in single cases only one assessment) of all assessments with BLUEs > 15% and Rep > 0.4 were used. Special attention was paid on the analysis of percentage data. Model fits often resulted in non-normally distributed genotype means (right skewed), increasing residual variance with increasing scores and negative BLUEs and thus it was decided to use a generalized mixed linear model (GLMM). We chose a logit link-function with binomial variance. Ideally not only the link function but also the variance function can be specified in the model calculation function, but to the best of our knowledge it was not possible to fit with ASReml-R 4.1.0.160 (Butler 2021). However, by default the asreml function with the argument “family = list(asr_binomial(link = “logit”, dispersion = NA))” calculates overdispersion and directly corrects for it. Except this additional argument in the function call, data were modeled likewise to a mixed model with normal link function. Outliers were detected by using method 2 (Bonferroni-Holm using studentized residuals) described in Bernal-Vasquez et al. (2016), where for the GLMM the deviance residuals were used.

#### Second stage

BLUEs ${g}_{tij}$ with standard errors from the first stage were used to calculate BLUEs ${g}_{i}$ across environments by

2 $$g_{tij} = \mu + g_{i} + l_{t} + gl_{it} + e_{tij} ,$$

with effects for the *t*th environment l, the genotype-environment interaction ${gl}_{it}$ and the residual error e. To account for the errors from the first stage the reciprocal of the squared standard error of the BLUES was used as weights (method 2, Möhring and Piepho 2009). Consequently, the error variance of the residual was restricted to 1. To estimate variances, all effects were fitted as random. To estimate BLUEs and $\overline{v }$ for heritability calculation (*H*^*2*^ = *Rep*), the genotype was fitted as fixed effect.

Despite the advantages of the GLMM analysis, BLUEs for SR and YR were also back-transformed on a percentage scale to better compare them with a previous study (Miedaner et al. 2020). Thus, values on percentage scale refer to back-transformed values, all statistics like mapping or correlations were based on the logit scale because error estimates cannot easily be back-transformed.

### Marker analysis

All material was genotyped by TraitGenetics (SGS Institut Fresenius GmbH, TraitGenetics Section, Seeland OT Gatersleben, Germany) using a 25 K Infinium iSelect arraySNP chip. Marker data from the biparental populations were filtered for minor allele frequency (maf) > 0.2, call rate (CR) > 0.95 and for heterozygosity < 0.05 in case of the DH populations (Pop2–Pop7). Based on marker data several genotypes appeared to be identical (correlation > 0.99) and each group of identical genotypes was considered a single genotype and this already for phenotypic data analysis. Additionally, 4, 2, 4, 2 and 4 genotypes were dropped due to average marker heterozygosity > 0.1 in the DH populations Pop2, Pop4, Pop5, Pop7 and Pop8, respectively. Heterozygosity was attributed to spontaneous cross pollination during seed multiplication. After filtering, marker data from the biparental populations were converted into ABH and numeric format (A = − 1 = allele from parent 1, B = 1 = allele from parent 2, H = 0 = heterozygous).

Validation of the presence of major SR resistance genes postulated in the study (*Sr24*, *Sr31*, *Sr38*) and absence of *Yr5*, *Yr10* and *Yr15* was done by PCR marker tests that detect the alien chromatin where the first three genes come from. Markers used were Sr24#12 (*Sr24*), Xbarc71 (*Sr2*4), iag95 (*Sr31*) and Ventriup-LN2 (*Sr38*) for SR and Yr5_ins (*Yr5*), ES1100 (*Yr10*) and barc8 (*Yr15*) for YR, respectively. Conditions for PCR and marker sequences for *iag95* were obtained from (https://maswheat.ucdavis.edu/) www.maswheat.ucdavis.edu as well as, from the GrainGenes database (https://wheat.pw.usda.gov). Differential lines harboring the respective resistance genes were used as positive controls, whereas Avocet S and Cartago served as negative controls.

### QTL mapping

Linkage mapping was based on regressing each marker on the BLUEs estimated in the second stage:

3 $${g}_{i}= \mu + \alpha {x}_{i}+{G}_{i}+{e}_{i},$$

where $\alpha$ denotes the regression coefficient of marker x coded for the allele of parent 1 (-1), the heterozygote (0) and parent 2 (1). The genetic variance was structured based on a kinship matrix K calculated from all markers by $K=Z{Z}{\prime}/2\sum {p}_{j}(1-{p}_{j})$, where Z is a n × m matrix with n genotypes and m markers scaled for the allele frequency ${z}_{ij}={x}_{ij}-2{p}_{j}$ and ${x}_{ij}$ the allele for genotype *i* and marker *j* of marker matrix M (VanRaden 2008). Like the second stage phenotypic model, the reciprocals of the squared standard errors of the BLUEs were used as weights in the regression model. After a first mapping run, single significant markers were fitted as fixed cofactors, but only if the distance was not smaller than 20 cM. Pairwise recombination R between markers m × m was estimated. For DH populations this was calculated by

4 $$R = \frac{{0.5\left( {\left| {M - 1} \right|\prime \left| {M - 1} \right| - \left( {M - 1} \right)\prime \left( {M - 1} \right)} \right)}}{{\left| {M - 1} \right|\prime \left| {M - 1} \right|}} \times 100$$

For each single marker fit, *P*-values were extracted from Wald-test statistics and to adjust for multiple testing the global significance threshold was calculated using the simpleM method (Gao et al. 2008), but instead of splitting the marker matrix into several chromosomes, singular value decomposition was applied for the whole marker matrix at once. The package RSpectra was used for computation (Qiu and Mei 2019). The explained genetic variance (pG) was calculated by the difference of estimated genetic variances of model 3 and model 2, divided by the genetic variance estimated by model 2.

## Results

### Phenotypic data

Both SR and YR data showed a pronounced right skew in the scoring data in all environments (Fig. S1). Thus, trait differentiation was small leading to low mean infection levels at the individual locations (Tables S3 and S4). Logit transformation was used throughout. In all cases the last scoring date showed the highest trait differentiation and hence also the highest estimated repeatability.

Genotypic variances were in all cases, except Pop6 SR resistance, larger than genotype-environment interaction variance (Tables S5 and S6). In the latter population both parents were moderately susceptible. Overall, the highest genetic variance was observed in Pop2 for SR and in Pop5 and Pop7 for YR. Entry-mean heritabilities ranged between 0.37 (Pop6) and 0.84 (Pop1) for SR and 0.58 (Pop6) and 0.90 (Pop1) for YR.

**Fig. 1:**
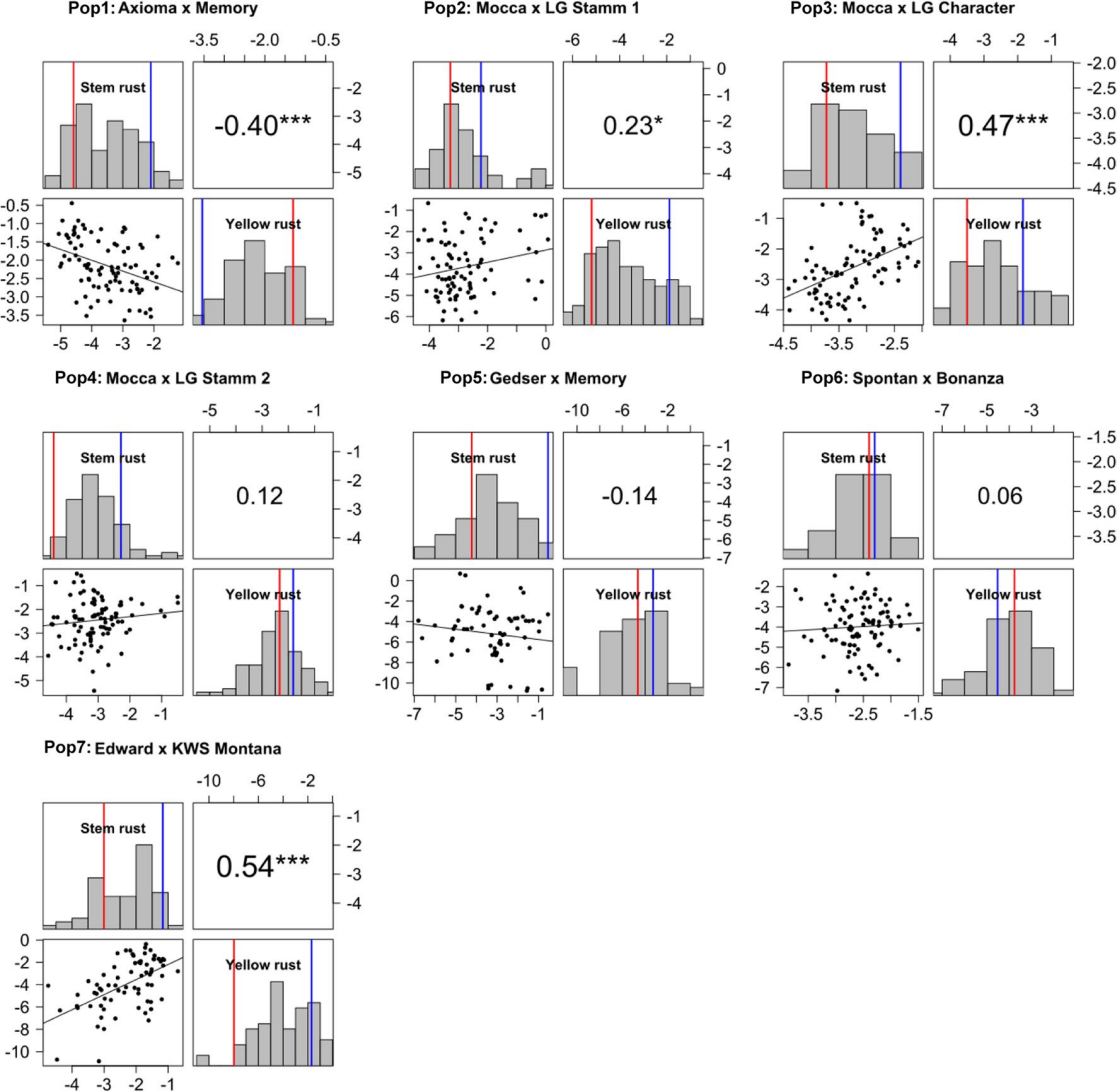
Correlation plot and histograms for yellow rust and stem rust responses in all populations based on best linear unbiased estimators (BLUEs) on logit scale. Blue line indicates BLUE of Parent 1, red lines of Parent 2. ***P < 0.001 alpha level (colour figure online)

After logit transformation, histograms displayed normal distributions (Fig. 1). Four of seven populations had a significant trait correlation between SR and YR, namely Pop1, Pop2, Pop3, and Pop7 (Fig. 1), although the correlation was negative in Pop1.

### Genotypic data and QTL mapping

**Fig. 2:**
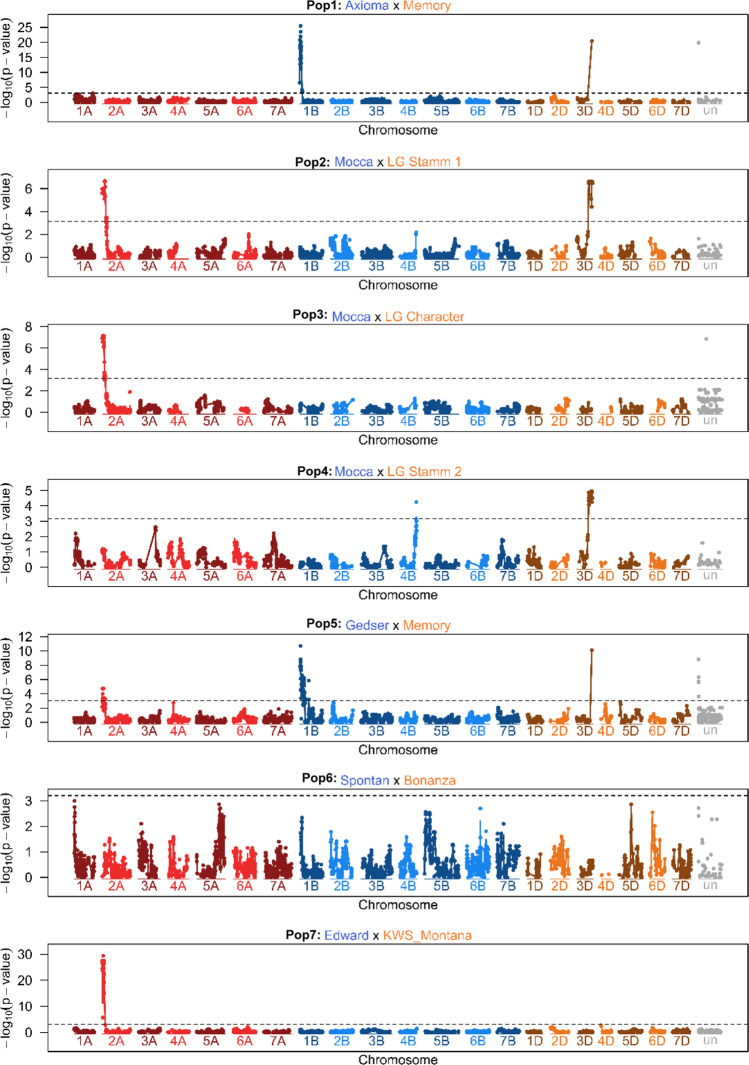
Manhattan plots of the phenotypic means for stem rust for the biparental populations (headers from top to bottom). In the Manhattan plot the − log_10_ of the *P*-value is displayed for all markers on the chromosomes of each genome according to a consensus map calculated from separate linkage maps based on marker data of the biparental populations. Markers that could not (uniquely) be assigned to a chromosome are displayed on the chromosome “un” with arbitrary positions. Manhattan plots of the biparental populations were interpolated with a spline function (colour figure online)

For SR, across all populations except Pop6 one to three major *Sr* genes were detected (Table 3, Fig. 2), namely *Sr24* on chromosome 3D (Pop2, Pop4, Pop5), *Sr31* on chromosome 1B (Pop1, Pop5), and *Sr38/Yr17* on chromosome 2A (Pop2, Pop3, Pop5, Pop7) either alone or in different combinations. Additionally, in Pop4 there was a major gene on chromosome 4B. One to two QTLs were additionally detected in Pop1, Pop2, and Pop4. They made only small contribution to SR response. All genes/QTLs together explained up to > 90% of the genetic variance summing in all populations. No QTL for SR response was found in Pop6. The postulated SR resistance genes were supported by additional PCR marker tests except for*Sr38/Yr17* in Pop6 (Table 5) where the respective SR gene was not detected by linkage mapping.

**Table 3:** Mapping results for stem rust resistance across environments (logit scale)

Marker	Chr	Pos. [bp] of marker	*P*-value	*P*-value cofactor	Eff	SE	*N*_Geno_ (allele)			pG
							0	1	2	
Pop1: Axioma × Memory										
Kukri_c2332_583	1A	7,660,991	1.1 × 10^–2^	2.1 × 10^–6^	− 0.8	0.3	40	25	23	0.12
Kukri_c36151_170 (*Sr31 or Sr1RS*^*Amigo*^)	1B	9,555,825	1.5 × 10^–21^	1.8 × 10^–17^	− 1.6	0.2	30	20	38	0.79
AX.158557688	2D	12,544,152	3.5 × 10^–2^	2.7 × 10^–4^	− 0.5	0.3	33	20	37	0.11
RAC875_c51595_177 (*Sr24*)	3D	613,700,556	3.6 × 10^–21^	1.8 × 10^–17^	− 1.6	0.2	33	22	34	0.80
Total										0.98
Pop2: Mocca × Stamm 1										
IAAV8501 (*Sr38/Yr17*)	2A	12,327,389	1.0 × 10^–6^	2.5 × 10^–8^	− 1.3	0.3	29		50	0.57
AX.158575237	2B	7,676,708	2.2 × 10^–2^	2.6 × 10^–3^	− 0.7	0.3	41	3	34	0.07
RAC875_c51595_177 (*Sr24*)	3D	613,700,556	2.8 × 10^–7^	1.1 × 10^–7^	− 1.2	0.2	43	2	34	0.58
BobWhite_c11808_975	6D	7,098,505	4.0 × 10^–2^	4.6 × 10^–4^	0.5	0.2	32		47	0.01
Total										1.00
Pop3: Mocca × LG Character										
IAAV8501 (*Sr38/Yr17*)	2A	12,327,389	7.4 × 10^–8^	3.1 × 10^–6^	− 0.9	0.2	34		51	1.00
Pop4: Mocca x LG Stamm 2										
AX.94779538	4B	665,452,409	5.7 × 10^–5^	5.6 × 10^–3^	0.8	0.2	45	1	38	1.00
RAC875_c51595_177 (*Sr24*)	3D	613,700,556	3.2 × 10^–5^	3.1 × 10^–3^	− 0.8	0.2	42		43	0.77
Total										1.00
Pop5: Gedser × Memory										
IAAV8501 (*Sr38/Yr17*)	2A	12,327,389	6.9 × 10^–3^	1.5 × 10^–4^	− 0.8	0.3	29		36	0.34
Kukri_c36151_170 (*Sr31 or Sr1RS*^*Amigo*^)	1B	9,555,825	1.4 × 10^–8^	5.3 × 10^–16^	− 1.7	0.3	39		25	0.52
RAC875_c51595_177 (*Sr24*)	3D	613,700,556	7.3 × 10^–11^	2.3 × 10^–18^	− 2.1	0.3	40	1	24	0.59
Total										0.98
Pop7: Edward × KWS Montana										
IAAV8501 (*Sr38/Yr17*)	2A	12,327,389	3.3 × 10^–28^	1.7 × 10^–17^	− 1.4	0.1	36		28	0.91

Chr., chromosome on consensus map; Pos., physical position in base pairs (bp) provided by the sequencing company; *P*-value, *P*-value from linear regression of the respective marker; *P*-value cofac, *P*-value from linear regression of the respective marker by using other markers as cofactors: Eff., Effect, estimated from marker regression as half of the difference between homozygous alleles, negative effect indicates that the allele is from parent 2 (Pop1-Pop7); SE, standard error of the effect estimate; *N*_Geno_, number of lines with the respective allele, pG, explained genetic variance

**Fig. 3:**
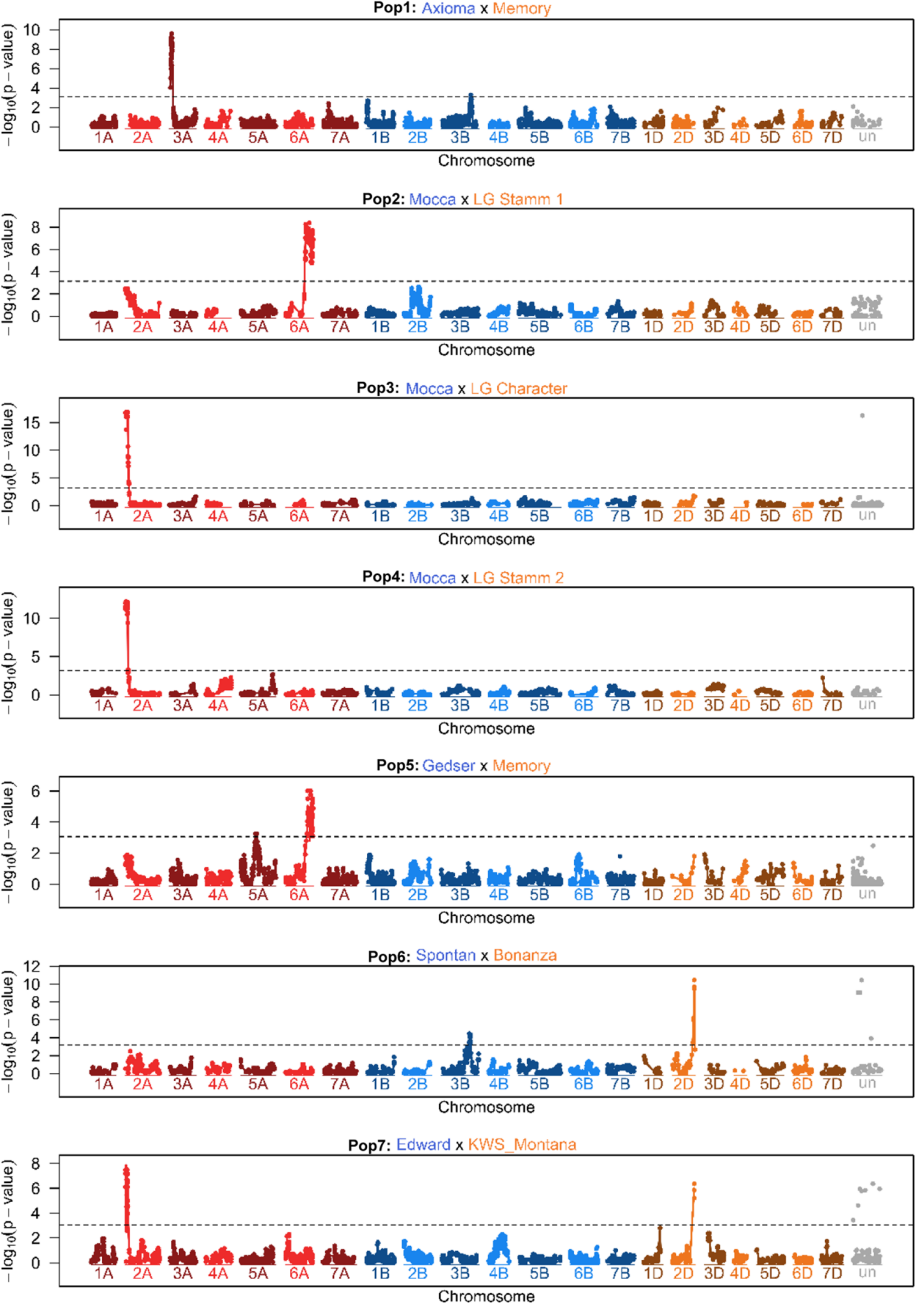
Manhattan plots of the phenotypic means for the trait yellow rust for the biparental populations (headers from top to bottom). In the Manhattan plot the − log_10_ of the *P*-value is displayed for all markers of each genome according to a consensus map calculated from separate linkage maps based on marker data of the biparental populations. Markers that could not (uniquely) be assigned to a chromosome are displayed on the chromosome “un” with arbitrary positions. Manhattan plots of the biparental populations were interpolated with a spline function (colour figure online)

For YR, two (Pop1) to four (Pop5) QTLs per population were identified across environments, with explained genetic variances ranging from about 1 to 99% indicating that both major QTLs/genes and minor QTLs were involved (Table 4, Fig. 3). In five populations, markers probably linked with *Sr38/Yr17* were detected on chromosome 2A. Their contribution to genotypic variance ranged from 13 to 99% depending on the number of QTLs detected in the same population (Table 4). Of the other QTLs, those on chromosome 3A (Pop1), 6A (Pop2, Pop5) and 2D (Pop5, Pop6, Pop7), made major contributions to genotypic variance in some populations. Additionally, there were eight minor QTLs for YR response. Markers linked with the known and (in Europe) still effective resistance genes *Yr5*, *Yr10*, and *Yr15* were not detected in any marker analysis.

**Table 4:** Mapping results for yellow rust resistance across environments (logit scale)

Marker	Chr	Pos. [bp]	*P*-value	*P*-value cofac	Eff	SE	*N*_Geno_ (Allele)			pG
							0	1	2	
Pop1: Axioma–Memory										
BS00073062_51	3A	12,997,745	7.3 × 10^–10^	7.6 × 10^–8^	1.1	0.2	44	19	27	0.59
Excalibur_c44713_137	3B	n.a	7.5 × 10^–2^	1.4 × 10^–3^	0.5	0.3	42	21	27	0.06
Total										0.70
Pop2: Mocca × Stamm 1										
IAAV8501 (*Sr38/Yr17*)	2A	12,327,389	9.2 × 10^–3^	2.1 × 10^–4^	− 0.9	0.3	29		50	0.16
GENE.4021_496	6A	610,349,860	2.7 × 10^–8^	2.0 × 10^–10^	− 1.7	0.3	32		46	0.61
Excalibur_c45094_602	2B	158,643,732	3.7 × 10^–3^	8.1 × 10^–7^	− 1.2	0.4	42	1	35	0.12
Total										1.00
Pop3: Mocca × LG Character										
IAAV8501 (*Sr38/Yr17*)	2A	12,327,389	8.6 × 10^–17^	2.0 × 10^–13^	− 1.4	0.2	34		51	0.99
Pop4: Mocca × Stamm 2										
IAAV8501 (*Sr38/Yr17*)	2A	12,327,389	2.0 × 10^–12^	2.3 × 10^–11^	− 1.3	0.2	34	2	48	0.88
wsnp_Ex_c23795_33033150	5A	679,665,894	2.3 × 10^–3^	8.4 × 10^–4^	0.7	0.2	36	2	47	0.15
Total										0.98
Pop5: Gedser × Memory										
IAAV8501 (*Sr38/Yr17*)	2A	12,327,389	1.6 × 10^–2^	1.6 × 10^–4^	− 1.4	0.6	29		36	0.13
AX.158558835	5A	479,600,503	5.0 × 10^–3^	3.4 × 10^–3^	1.8	0.6	30		34	0.16
GENE.4021_496	6A	610,349,860	9.7 × 10^–7^	4.6 × 10^–7^	2.4	0.5	41		24	0.47
TA017943.0495	2D	636,600,183	1.6 × 10^–2^	2.9 × 10^–4^	− 1.3	0.5	37		28	0.06
Total										0.77
Pop6: Spontan × Bonanza										
BS00040742_51	3B	741,304,140	3.5 × 10^–5^	1.9 × 10^–4^	− 1.0	0.2	40		51	0.34
AX.158557197	1D	9,255,864	1.5 × 10^–2^	3.2 × 10^–4^	− 0.5	0.2	48		41	0.18
TA017943.0495	2D	636,600,183	3.4 × 10^–10^	2.6 × 10^–9^	1.2	0.2	54		38	0.60
Total										0.92
Pop7: Edward × KWS Montana										
IAAV8501 (*Sr38/Yr17*)	2A	12,327,389	8.0 × 10^–7^	2.2 × 10^–6^	− 2.0	0.4	36		28	0.44
BS00068851_51	4B	527,956,801	5.0 × 10^–3^	2.7 × 10^–5^	− 1.5	0.5	32		32	0.14
TA017943.0495	2D	636,600,183	4.4 × 10^–7^	4.9 × 10^–8^	− 1.6	0.3	35	1	28	0.33
Total										0.81

Chr., chromosome on consensus map; Pos., physical position in base pairs (bp) provided by the sequencing company; *P*-value, *P*-value from linear regression of the respective marker; *P*-value cofac, *P*-value from linear regression of the respective marker by using other markers as cofactors: Eff., Effect, estimated from marker regression as half of the difference between homozygous alleles, negative effect indicates that the allele is from parent 2 (Pop1-Pop7); SE, standard error of the effect estimate; *N*_Geno_, number of lines with the respective allele, pG, = explained genetic variance

### Effects of individual and combined genes/QTL

**Fig. 4:**
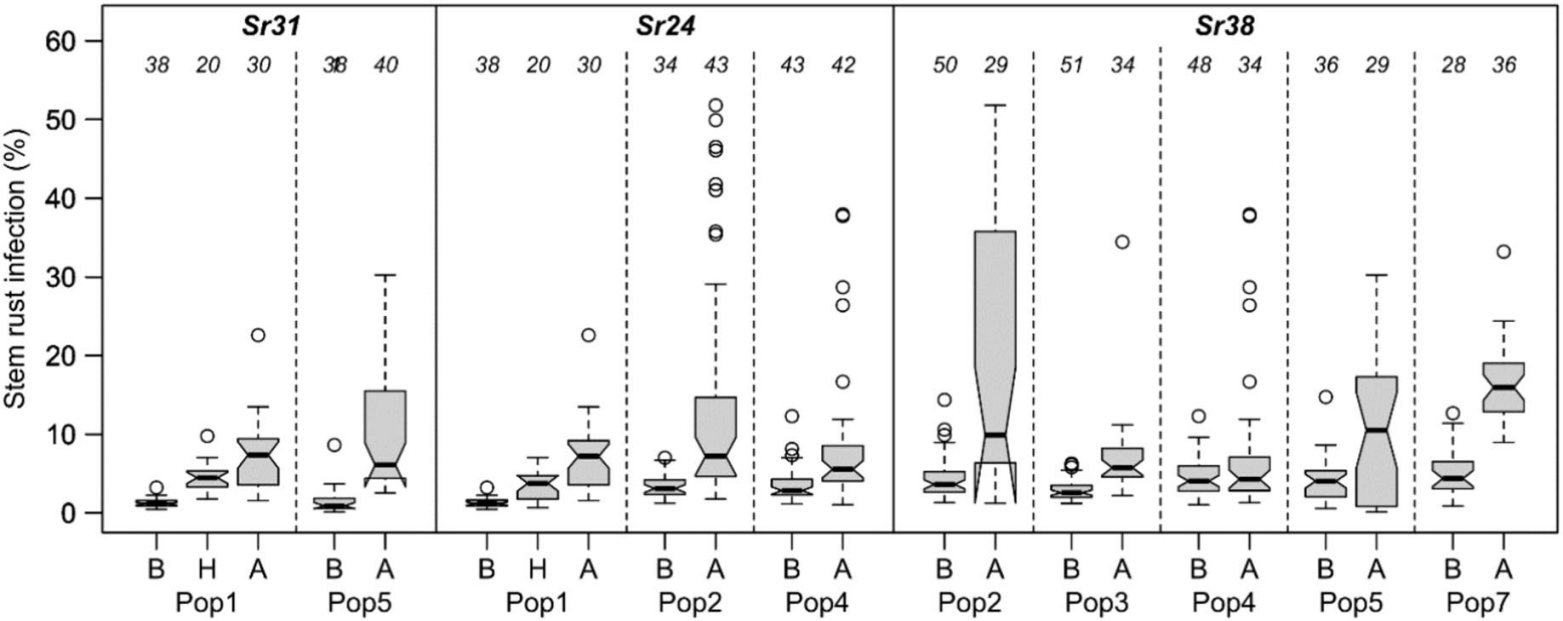
Boxplots of genotypes grouped by alleles of markers linked with three stem rust (*Sr*) genes in the respective populations. Allele A indicates the allele of parent1 and allele B of parent2. Number of genotypes in the respective group are reported above boxes. Genotypes with missing marker alleles were not included

**Fig. 5:**
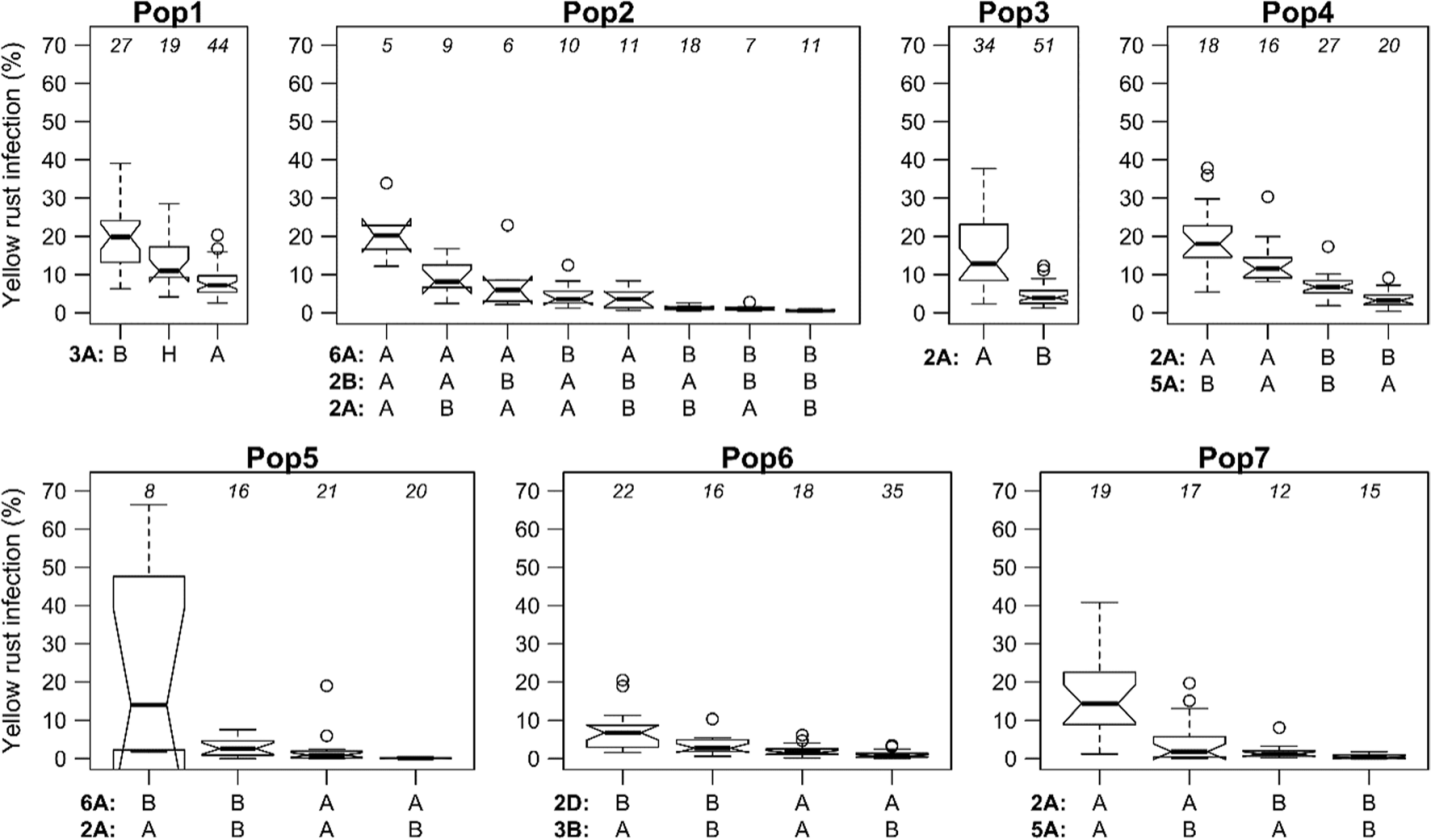
Boxplots of genotypes grouped for individual and combined alleles of different genes/QTL found for yellow rust resistance in seven populations. The alleles and allele combinations of respective groups are reported on the *x*-axis. Allele A belongs to the allele of parent 1, allele B to parent 2 and H to heterozygous. As only single QTLs were found on separate chromosomes, chromosome names can be used to deduce respective markers used for clustering from Table 4. To keep reasonable group sizes, for some populations only two or three most significant QTLs were used for clustering. Numbers of genotypes in respective group are reported above the boxes

*Sr31* and *Sr24* had large effects on SR response in both populations where they showed no overlapping 50% quantiles between plants with the resistant and the susceptible allele (Fig. 4). This was also the case for *Sr38/Yr17* in three populations (Pop2, Pop3, Pop7), but not in the remaining two (Pop4, Pop5). For YR, the multiple resistance alleles in each population except for Pop 7 were near-additive. Where two genes/QTL had large effects the disease responses approached zero (Fig. 5). In four populations two resistance-associated markers were enough to reduce YR severity to zero.

**Fig. 6:**
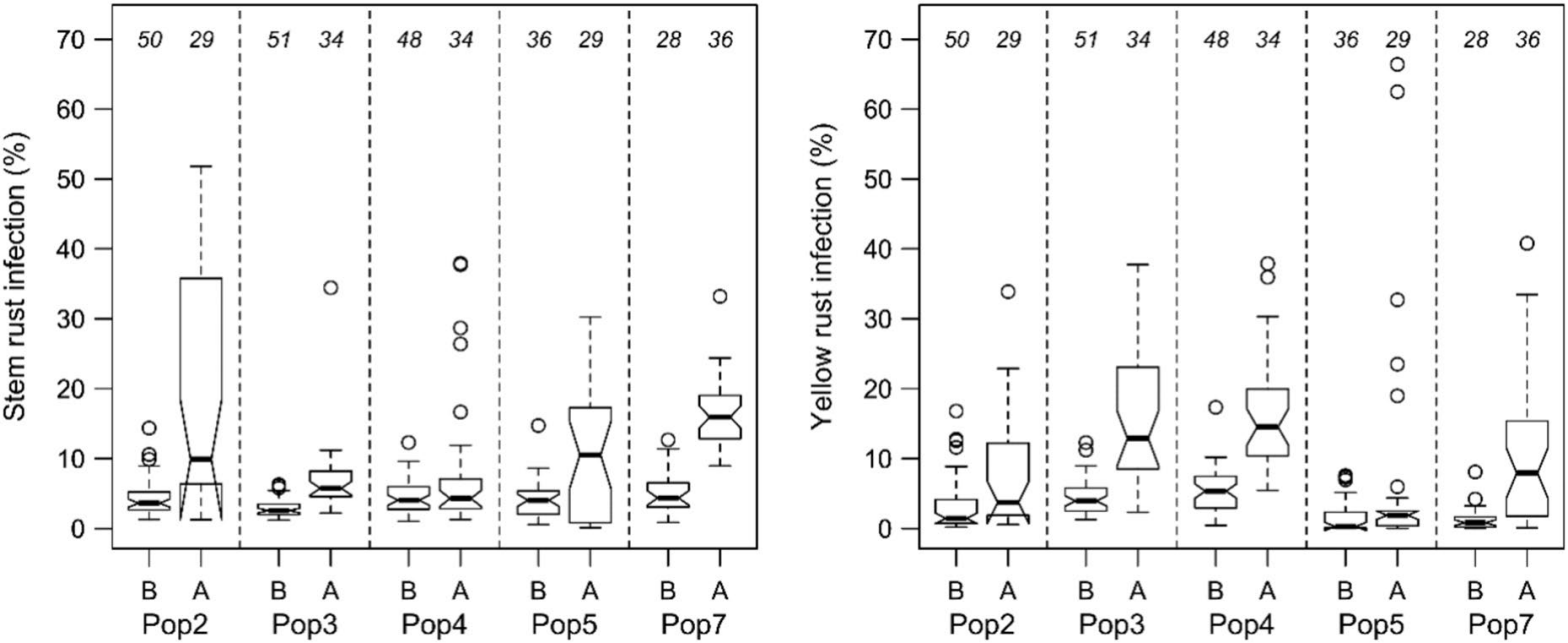
Boxplots of genotypes grouped by alleles of marker IAAV8501 linked to *Sr38/Yr17* on chromosome 2A for stem rust and yellow rust response analyzed as best linear unbiased estimators (BLUEs) for five populations (separated by dashed lines). Allele A indicates the allele of Parent 1, allele B of Parent 2. Numbers of genotypes in respective groups are reported above boxes. Genotypes with missing marker alleles were not included

The *Sr38/Yr17* locus resulting in partial resistance to both YR and SR had very different effects depending on the cross (Fig. 6). In Pop3 and Pop7, the effect was large for both diseases, whereas in Pop4 the effect was only large for YR and in Pop2 only for SR. No significant effect for YR response was detected in Pop5.

## Discussion

### Methodological aspects

Multiple YR epidemics in Central Europe from 2014 to 2016, caused by the emergence of the *Pst* Warrior race, led to numerous mapping studies (e.g., Beukert et al. 2020; Shahinnia et al. 2022; Bouvet et al. 2022b; Lin et al. 2023). However, this is the first study to examine the inheritance of both SR and YR resistances in segregating populations. The populations provided by three plant breeding companies represent current breeding material. In contrast to other studies, we used artificial inoculation by individual *Pst* and *Pgt* races selected from the current race composition in Central Europe (GRRC 2023a,b). Although the disease responses were evaluated at the adult-plant stage, we also tested the parental lines at the seedling stage (Table 2). Thus, in the field each population was inoculated with the isolate showing the most differentiation based on the seedling test. For Pop1, we used race HFCLB in 2020 and race TKTTF in 2021; both races showed similar responses with Axioma being susceptible and Memory being resistant (Table 2). Especially for yellow rust natural infection with other races was possible but we believe this had little effect.

In general, population sizes were small, but in this study we expected mainly major resistance genes. With small population size the resolution of QTL mapping is limited and large linkage blocks lead to a high number of redundant markers and limited precision of estimated QTL positions (Xu 2003; Beavis 1998). A small population size also limits the detection of a major gene when other major genes are available in the same germplasm. Nonetheless, in contrast to genome-wide association studies (GWAS), biparental QTL studies have a higher QTL detection power even at lower marker densities due to balanced allele frequencies. In addition, common markers for the alien chromatin from which the SR resistances are derived were used to verify the resistances across parents (Table 3 and 4). Throughout this study, major QTLs are defined to have explained genetic variance p_G_ > 25%.

Marker-based studies cannot prove the identity of a linked marker with a known resistance gene, at least until the resistance gene is cloned. Only the physical location of the two loci can be compared and this is what is done below. The new ‘Genome Atlas’ for rust resistance loci (Tong et al. 2024) does not help in this respect, but for the first time, physical positions are given for 920 leaf rust, yellow rust, and stem rust resistance genes/QTLs allowing a better comparison among studies. Concerning older QTL publications it should be noted that their precision is about 10 Mbp (Tong et al. 2024). Another problem in this respect could be that the linked marker(s) might not remain in the same LD block with the causal gene across generations due to recombination, thus comparison among studies also gets difficult (Tong et al. 2024). We could circumvent this problem in some cases by addressing SR genes from alien introgression events where PCR-based markers are available for the detection of the introgressed segment (Table 5).

### All-stage versus adult-plant resistance in the parents

For the release of cultivars in Central Europe only adult-plant data are recorded. Seedling tests are an important tool for breeders to accelerate selection of resistance if they desire, but more important to dissociate all stage resistance from adult plant resistance with its reputed higher durability. We selected the parents in this study based on seedling tests especially those resistant to both YR and SR (Memory, Stamm 1, Stamm 2, LG Character, Spontan) or only to YR (Axioma, Bonanza, KWS Montana). Our adult-plant stage results largely paralleled the seedling results, with only Spontan proving to be moderately susceptible to SR and KWS Montana more resistant to SR than predicted from seedling tests.

In a previous study, no *Sr* gene was postulated for Memory because it was resistant to all isolates from a worldwide collection including the highly virulent races TTKSK, TRTTF, TKTTF, TTTTF (Flath et al. 2018). Hence, Memory was included in the present study twice. Its pedigree is complex and resistant parents like Kronjuwel and Amigo, but also Piko, Atlantis, and Cardos are included (Kempf, pers. commun.). Amigo is a 1AL/1RS translocation with *Sr1RS*^*Amigo*^, *Pm17* and, in some plants, *Lr24* (McIntosh et al. 1995). However, wheat cultivars Kronjuwel and Atlantis have the 1BL/1RS translocation (http://wheatpedigree.net/) and Kronjuwel is reported to have *Sr31* (Porceddu et al. 1988). As we identified a QTL for SR resistance on chromosomes 1A and 1B in Pop1, we cannot decide whether *Sr1RS*^*Amigo*^ or *Sr31* is present in Memory because the marker *iag95* identifies rye chromatin. However, the 1B QTL explained a much higher proportion of explained genotypic variance than the 1A QTL (79% vs. 12%, Table 3). According to our results, Memory contains all three *Sr* genes (*Sr38, Sr31* or *Sr1RS*^*Amigo*^, and *Sr24*) as shown from Pop5 and supported by our PCR marker analysis, and additionally four QTLs explaining 11 to 52% of genotypic variation. This and the occurrence of the additional QTLs explain why no *Sr* gene could be postulated for Memory in the earlier study by Flath et al. (2018). In Pop1, *Sr38* was not detected in Memory by mapping, because the other parent Axioma carried the same gene (Table 5). In the field test, Memory showed only 1.0% and 1.4% SR severity, respectively, whereas the most susceptible cultivar, Gedser, had 37% severity. An example of an adult-plant SR resistance source might be the parent KWS Montana, which was susceptible (IT 5) at the seedling stage, but displayed only 5% severity in the field.

**Table 5:** Tests of resistant parents by PCR markers for identified stem rust resistance genes

Population	Parents	*Sr24*	*Sr24*	*Sr31 or Sr1RS*^*Amigo*^	*Sr38*
	Primer →	Sr24#12	Xbarc71	Iag95	Ventriup-LN2
Pop1	Axioma	**−**	**−**	**−**	**+**
	Memory	**+**	**+**	**+**	**+**
Pop2	LG Stamm1	**+**	**+**	**−**	**+**
Pop3	LG Character	**−**	**−**	**−**	**+**
Pop4	LG Stamm2	**+**	**+**	**−**	**+**
Pop5	Memory	**+**	**+**	**+**	**+**
Pop6	Spontan	**−**	**−**	**−**	**+**
Pop7	KWS Montana	**−**	**−**	**−**	**+**

For YR, all parents except Mocca and Edward were resistant or moderately resistant at seedling and adult-plant stages (Table 2). Gedser was seedling-susceptible, but moderately resistant in the field. Compared to field trials, Memory was resistant in Pop5, but rather susceptible in Pop1 (Table 2). The latter might be caused by the extremely high infection pressure at the LEM location in both years (Table S4).

### Three major SR genes present in the tested breeding material

Across all populations, there were three major QTL on chromosomes 2A, 1B and 3D for SR resistance. The physical positions reported for these three genes corresponded to alien chromosome segments bearing *Sr38/Yr17/Lr37* (chromosome 2A), *Sr31* (chromosome 1B), and *Sr24* (chromosome 3D). We also verified the respective alien segments in the parental lines (Table 5). It was already known that these three genes are present in different combinations in German and Czech winter wheat varieties (Flath et al. 2018; Zelba et al. 2022). Six SR resistance QTLs that were not overlapping between populations were also found.

*Sr24* derived from *Thinopyron ponticum* was located on chromosome 3D (McIntosh et al. 1977). Walkowiak et al. (2020) reported the *Th. ponticum* introgression segment on the long arm of 3D to have a size of approximately 60 Mbp. Mago et al. (2005a) developed several markers for mapping of *Sr24*. Using primer sequence Sr24#12 showed the presence of this gene in Memory, LG Stamm 1, and LG Stamm 2 (Table 5). *Sr24* was frequently used in Australian, South and North American and South African breeding material (Jin et al. 2008). The presence of *Sr24* in European breeding material is due to selection of leaf rust resistance gene *Lr24* (Flath et al. 2018).

*Sr31* and/or *Sr1RS*^*Amigo*^ was present in two populations (Pop1, Pop5) with the same, most significant marker located at 9.56 Mbp on the physical map and verified by PCR-based marker *Iag95-STS* (Mago et al. 2002). Both genes were derived from wheat-rye translocations including additional resistance genes against leaf rust (*Lr26*), yellow rust (*Yr9*) and powdery mildew (*Pm8*) (Mago et al. 2005b; Ren et al. 2009). Due to the extensive use of the yield-enhancing wheat-rye translocation in European wheat, *Sr31* is frequently present in wheat varieties by chance. *Sr31* was effective against all isolates collected during the local stem rust epidemic in 2013 in Central Germany, where in total six different races (TKTTF, TKKTF, TKPTF, TKKTP, PKPTF, MMMTF) were detected from only 17 samples that also differed in molecular markers (Olivera Firpo et al. 2017).

Interestingly, we detected a fourth prominent QTL for stem-rust resistance on chromosome 4B in the Mocca × LG Stamm 2 population with a physical position at 665.5 Mbp and of high explained genetic variance, however, the *P*-value was rather high (Table 3). The metaQTL study by Pal et al. (2022) found two MQTLs for stem rust resistance in the same region (4B.3 at 671.7–677.9 Mbp and 4B.6 at 660.7–663.1 Mbp) that were based on four and two QTLs, respectively. This QTL should get more attention by further fine-mapping and validation in different genomic backgrounds. In future, it should be determined if this QTL is *Sr8*.

### Major resistance cluster *Sr38/Yr17* provides resistance to stem rust and yellow rust

The major QTL on chromosome 2A found in Pop2, Pop3, Pop5, Pop7 for both SR and YR resistances and additionally in Pop4 for YR resistance corresponds to the rust resistance gene cluster *Sr38/Yr17/Lr37* introgressed from *Aegilops ventricosa* chromosome arm 2NS (Seah et al. 2001; Helguera et al. 2003).

For Pop2, Pop3, and Pop4 we used a *Pgt* isolate that was avirulent for *Sr38* (WSR-55/13-8, Table S2), however, the isolate inoculated on Pop5, Pop6, and Pop7 was virulent for this gene and we still detected *Sr38* in Pop5 and Pop7. The resistant parents inoculated with this isolate reacted in seedling stage either resistant (Memory), moderately resistant (Spontan) or even susceptible (KWS Montana) to this race. We could not detect this gene in Spontan (Pop6) where no SR resistance QTL was detected, probably due to low disease development. In Pop1 we could not detect this gene by QTL mapping because both parents carry it as shown by the PCR-based markers (Table 5).

The fact that *Sr38* still shows SR resistance at the adult plant stage in field trials, even though the inoculated SR races were virulent for this gene, has been described several times. In the study of Zhang et al. (2014), *Sr38* was the most effective gene in the field test in the USA. Nine cultivars with *Sr38* displayed strong resistance (0.55–3.42% SR severity) although a high virulence frequency for this gene was found in the inoculated *Pgt* population. In Europe, Flath et al. (2018) found that varieties with *Sr38* in the field had a SR severity of 0.6–16%, while the most susceptible variety had a value of 41.5%. Similarly, Zelba et al. (2022) reported that varieties with only *Sr38* had a field resistance of 3.2 on a scale of 1–9, although most pathotypes were virulent for it, while varieties without any *Sr* gene had 7.1. Obviously, a residual resistance for this gene is still effective in the adult-plant stage in terms of reduced infection as it has also been found for the SR resistance genes *Sr6*, *Sr8* and *Sr9a* (Brodny et al. 1986).

The yellow rust resistance gene *Yr17* is known to be overcome by several races of *Pst* in the adult-plant stage, at least by conducting seedling tests (Bayles et al. 2000)*.* Pop2, Pop3, Pop4, and Pop7 showing this gene were inoculated with isolates of the original Warrior race (*PstS7*) and the Warrior (–) race (*PstS10*) ‘Benchmark’, which were assessed as virulent to *Yr17* (Table S4). Although *Yr17* was described as all-stage resistance the decision to assess a *Pst* isolate as avirulent or virulent can be arbitrary based on the differential line, test environment and assessor and more recent publications by Liu et al. (2020) and Li et al. (2023) showing a second gene for APR in lines with the 2NS-2A translocation. The question then is whether the adult plant resistance phenotype is due to residual effects of *Yr17* or to the second gene. All populations had mean YR response levels ranging from 0.6 to 10.5% (Table S4). Milus et al. (2015) observed that some genotypes with *Yr17* were susceptible to *Pst* at the seedling stage but reached medium to highly resistant infection types during adult-plant stage. They also observed that only partially virulent isolates during seedling stage were not able to cause disease on adult plants in the field, but this was prior to current evidence that lines with the translocation had an additional resistance gene. This gives a further explanation why lines with the 2A-2NS translocation still contributed a significant resistance effect during adult plant stage and hence was mapped in this study. Such residual resistance was described for several defeated YR resistance genes including *Yr17* (Singh et al. 2022). In summary, *Sr38/Yr17* was present in all resistant parents investigated in our study (Table 5).

Phenotypically, we found positive significant correlations between YR and SR resistances in Pop2, Pop3 and Pop7 (Fig. 1). No significant phenotypic correlation was found in Pop4 and Pop5 where the *Sr38/Yr17* markers had been detected. However, correlation was likely masked by *Sr24* or other YR resistance QTLs, especially in Pop5 where the genetic variance explained by *Yr17* was 13%.

### Known genes for YR resistance

Five different major YR QTLs were mapped on chromosomes 2A, 3A, 6A, 3B, and 2D. Additionally, seven population-specific, minor effect QTLs for YR were detected across six chromosomes. The genes with major effects described here might already be mapped in other wheat populations given the high number of reported QTLs for this pathosystem (Pal et al. 2022; Kumar et al. 2023; Tong et al. 2024). The major genes *Yr10* and *Yr15* on chromosome arm 1BS that are widely distributed in some parts of the world (e.g., Kazakhstan, Kokhmetova et al. 2021) were not detected in this study. The same was true for *Yr5*. Although we found a QTL on chromosome 2B in Pop 2, its physical position of 158.6 Mbp was quite distal to the position of *Yr5* (110.2–110.9 Mbp, Kumar et al. 2023).

A QTL for YR resistance was detected on chromosome arm 6AL in Pop2 and Pop5 with an explained genetic variance of 61% and 47%, respectively. This coincides well with a minor QTL associated with marker wsnp_Ex_rep_c101766_87073440on 6A reported by Bouvet et al. (2022b). This marker was also present on the 25 k chip used in this study but monomorphic in the populations segregating for the 6A-QTL. A QTL at approximately the same position was mapped by Cheng et al. (2022). Wang et al. (2021) placed the same locus at 609.38 Mbp and found a second QTL for seedling response at 595.67 Mbp in Chinese landraces. Both studies classified the QTL to be new and effective during all growth stages (ASR). Miedaner et al. (2020), Beukert et al (2020), Rollar et al. (2021), Shahinnia et al. (2022), Kale et al. (2022), and Lin et al. (2023) detected resistance QTLs at a similar position. All mapping studies placed this 6A QTL in a small interval ranging from 598 to 612 Mbp, encompassing the position of our most closely linked marker at 610.35 Mbp. Lin et al. (2023) found the same linked marker (GENE 4021_496 at 610 Mbp) and identified 18 annotated disease resistance genes in a ± 1 Mbp interval around this marker. However, none of the genes cloned in this segment code for a NLR motif (Hafeez et al. 2021). In summary, the 6AL QTL is an environmentally highly stable YR resistance gene that is frequent in modern wheat breeding materials (Lin et al. 2023) comprising a good field resistance. It is yet not clear whether one or more genes are responsible and whether it is a ASR or APR locus. In future, it would be worthwhile to clone this gene from one source and use the sequence for analyzing the other populations.

A major QTL on chromosome 3A was mapped at 13 Mbp in Pop1. Bouvet et al. (2022b) reported a major QTL for YR on chromosome arm 3AS in a MAGIC mapping population that captured > 80% of the genetic variation in UK wheat. The physical position of the peak SNP marker in our study was about 0.77 Mbp distant from the position (Kukri_c28650_111, 7.921 Mbp) mapped by Bouvet et al. (2022b). This marker was on the 25 k chip used for genotyping in this study but was monomorphic in Pop1.

The major QTL on chromosome 3B was mapped at 741.3 Mbp in Pop6. The minor QTL on the same chromosome in Pop1 is unlikely to be the same. Wang et al. (2021) found a QTL for APR between 739.04 and 743.51 Mbp on chromosome 3B, as well as an ASR-associated QTL at 772.47 Mbp. Further research e.g., using fine-mapping approaches with increased population sizes, is needed to determine if the major QTL of our study coincides with already known APR resistance gene *Yr80*.

Another QTL for YR was mapped on the long arm of chromosome 2D at 636.60 Mbp in Pop6 and Pop7. In Pop5 the same marker made only a minor contribution to YR resistance. Bouvet et al. (2022b) found a YR QTL at 638.38 Mbp on chromosome 2D (Ra_c21099_1781). *Yr54* is known to be distally located on chromosome 2D (Basnet et al. 2014) and may coincide with a major YR QTL found by Jagger et al. (2011) in the German variety Alcedo, although they have no known ancestral relationship (Basnet et al. 2014). Marker *Xgwm301* 0.5 cM from APR gene *Yr54* apart that Basnet et al. (2014) located at ~ 648.88 Mbp falls into the interval of significant markers in the present study. In Basnet et al. (2014) *Yr54* explained 49–54% of the phenotypic variation. Another resistance gene, *Yr55,* is also located on 2D at 614.15 Mbp, and linked to marker *Xmag4089* (Xue et al. 2008; McIntosh et al. 2014). Hence it is possible that the QTL in our study corresponds to either of the two known YR resistance genes.

## Conclusions

YR resistance in our study was inherited in a quantitative manner by several QTL with major or minor effects. Most populations had one major QTL and one to three minor QTL segregating for YR resistance adding to 12 loci in seven populations. The diversity of resistance genes in the few parents in our study is likely underestimated due to the small population size, but might explain why 73% of the seed multiplication area in Germany includes varieties that are resistant to YR (score 1–3 on the 1–9 scale, where 1 = totally resistant, BSL 2024). Each cross analyzed here segregated at several loci. The combined action of these genes many of which conferred APR with reputed durability is expected to confer more durability.

The durability of the three mapped SR resistance genes is questionable. *Sr24* is known to be effective against many *Pgt* races including the original *Ug99* (TTKSK) but has been overcome by *Ug99* variants such as TTKST (Jin et al. 2007) as well as by race TKKTP in the 2013 German epidemic (Olivera Firpo et al. 2017; Flath et al. 2018). Hence it is still conferring resistance in Europe but should be considered vulnerable wherever cultivars with this gene are grown. *Sr31* is still a valuable resistance source in Europe but heed must be taken from the Ug99 events and the recent warning that virulent races could emerge from sexual hybridization (Olivera et al. 2022; Patpour et al. 2022). However, virulence surveys always focus on seedling resistance, whereas breeders score resistances only in the adult-plant stage within their field trials. A good example of this study is the *Sr38*/*Yr17/Lr37* gene cluster, where we could detect partial but effective disease reduction in the field for both rust diseases although according to virulence studies it should have been overcome by some of the rust races we inoculated.

With the threat of increasing environmental temperatures and the recent outbreaks of stem rust in Europe there is a need to have defined resistance sources for the region. This study clearly shows that the resistance variation in a selected group of current German varieties is limited to three or four all-stage resistance genes that are vulnerable to virulence changes in the pathogen population. It will be necessary to look beyond European winter wheat germplasm for resistance sources, either by identifying and transferring resistance from related species (resistance that is most likely to be ASR, because of the technical difficulties involved) or sourcing resistant materials from other countries or programs, the most obvious of which is CIMMYT. Wheat with durable stem rust resistance largely based on the classic APR gene *Sr2* (or ‘Sr2 complex’ that still remains ill-defined). There are also so-called multi-pathogen resistance genes *Lr34*+, *Lr46*+and *Lr67*+ for which repeated analyses have identified small QTL effects on stem rust response. Each of these genes have added morphological effects such a pseudo black chaff and excessive leaf tip necrosis that breeders will need to address, but if durable resistance is to be a national objective they are the currently best understood targets with well proven genetic markers to support their exploitation.

Yellow rust resistance is a different issue—almost every quantitative genetics study worldwide has shown that any acceptable level of APR (e.g., MR or below in repeated tests) is based on the additive effects of a few chromosomal regions that either have common genes or clusters of multiple genes such the chromosome 6AL effect discussed above. Moreover, if heed is taken of the above multi-pathogen resistance genes there will be the added benefits coming from those genes. While for stem rust, new resistance sources must be urgently introgressed in European wheat breeding, for yellow rust, marker-based selection techniques like genomic selection (GS) might be a more efficient approach to accumulate minor and major resistance QTLs in single genotypes. Still, with resistances from more distant germplasm field trials remain necessary in the adult-plant stage and are the gold standard for resistance data generation.

## Supplementary Information

Below is the link to the electronic supplementary material.

Supplementary file1 (DOCX 168 KB)
