# Supplementary material for: Mapping rust resistance in European winter wheat: many QTLs for yellow rust resistance, but only a few well characterized genes for stem rust resistance
Source: Theor Appl Genet. 2024 Sep 5;137(9):215. doi: 10.1007/s00122-024-04731-9 (PMC11377555; doi:10.1007/s00122-024-04731-9)
Supplement: Supplementary file 1 — Supplementary file1 (DOCX 168 KB) [file 122_2024_4731_MOESM1_ESM.docx]

**Mapping rust resistance in European winter wheat: Many QTLs for yellow rust, but only a few well characterized loci in stem rust**

Thomas Miedaner, Wera Eckhoff, Kerstin Flath, Anne-Kristin Schmitt, Philipp Schulz, Johannes Schacht, Philipp Boeven, Wessam Akel, Hubert Kempf, Paul Gruner

**Supplementary files**

Table S1 Phenotyping of Pop1-Pop7 for yellow rust (YR) and stem rust (SR)

| Pop |  | 2020 | | | |  | 2021 | | | | |
| --- | --- | --- | --- | --- | --- | --- | --- | --- | --- | --- | --- |
|  |  | DAH | HOH | LEM | ROS |  | DAH | HOH | LEM | ROS | SOL |
| Pop1 |  | YR/SR | YR/SR | YR |  |  | YR | YR/SR | YR |  |  |
| Pop2-4 |  |  | YR/SR |  | YR |  |  | YR/SR |  | YR/SR |  |
| Pop5-7 |  |  |  |  |  |  | YR/SR | YR/SR |  |  | YR/SR |

DAH=Berlin-Dahlem, HOH=Stuttgart-Hohenheim, LEM=Lemgo, ROS=Rosenthal-Peine, SOL=Söllingen

**Table S2** Origin, denomination, virulence and genetic cluster of the isolates of *Puccinia graminis* f. sp. *tritici* (Pgt), *Puccinia striiformis* (Pst) that were used for inoculation of field trials

| Patho-gen | Material | Genetic group | Race | Isolate ID | | Origin location  (Federal State^1)^) | Sam-pling year | Virulences |
| --- | --- | --- | --- | --- | --- | --- | --- | --- |
|  |  |  |  | Lab | Reference |  |  |  |
| *Pgt* | Pop1 (2020) | Clade VIII | HFCLB | WSR-55/13-8 | WSR-55/13-8^2)^ | Söllingen (NI) | 2013 | *Sr5, 21, 7b, 8a, 9g, 17, 9a, 10, McN* |
|  | Pop1 (2021) | Clade IV-A.1 | TKTTF | WSR-B5/19-2 | DE09_21^2)^ | Silstedt (SA), (Barberry) | 2019 | *Sr5*, *6*, *7b*, *8a*, *9a*, *9b*, *9d*, *9e*, *9g*, *10*, *17*, *21*, *30*, *36*, *38*, *Tmp,* *McN* |
|  | Pop2-Pop4 | Clade VIII | HFCLB | WSR-55/13-8 | WSR-55/13-8^2)^ | Söllingen (NI) | 2013 | *Sr5, 21, 7b, 8a, 9g, 17, 9a, 10, McN* |
|  | Pop5-Pop7 | Clade IV-A.1 | TKTTF | WSR-B5/19-2 | DE09_21^2)^ | Silstedt (SA), (Barberry) | 2019 | *Sr5*, *6*, *7b*, *8a*, *9a*, *9b*, *9d*, *9e*, *9g*, *10*, *17*, *21*, *30*, *36*, *38*, *Tmp,* *McN* |
| *Pst* | Pop1 | PstS7 | Warrior | GR-11/19 | DE2019Kl_YR2^2)3)^ | Denzlingen (BW) | 2019 | *Yr1, 2, 3, 4, 6, 7, 9, 17, 25, 32, Sp, AvS, Amb* |
|  | Pop2-Pop4 | PstS10 | Benchmark | GR-217/16 | ^3)^ | Granskevitz (MV) | 2016 | *Yr1, 2, 3, 4, 6, 7, 9, 17, 25, 27, 32, Sp, AvS, Amb* |
|  | Pop5-Pop7 | PstS7 | Warrior | GR-166/16 | ^3)^ | Dornburg (TH) | 2016 | *Yr1, 2, 3, 4, 6, 7, 8, 9, 17, 25, 27, 32, Sp, AvS, Amb* |

^1)^ Federal state: SA = Saxony-Anhalt, TH = Thuringia, NI = Lower Saxony, MV = Mecklenburg-Western Pomerania, BW = Baden-Wuerttemberg

^2)^ Tested by GRRC, Global Rust Reference Center, Aarhus University, Denmark

^3 )^ Tested by Julius Kühn-Institute, Kleinmachnow, Germany

**Table S3** Mean, minimum and maximum of stem rust severity (%), heritabilities (H^2^) and Repeatabilities (Rep.) across environments (env.) and at the single locations, respectively (BLUEs, backtransformed)

| **Pop** | **Env.** | **Mean** | **Minimum** | **Maximum** | **H^2^/Rep.** |
| --- | --- | --- | --- | --- | --- |
| **Pop1** | **Across env.** | **2.9** | **0.5** | **22.6** | **0.84** |
| Pop1 | DAL_2020 | 2.5 | 0.0 | 23.5 | 0.59 |
| Pop1 | HOH_2020 | 5.0 | 0.5 | 33.8 | 0.90 |
| Pop1 | HOH_2021 | 0.2 | 0.0 | 28.9 | 0.09 |
| **Pop2** | **Across env.** | **6.0** | **1.2** | **51.8** | **0.83** |
| Pop2 | HOH_2020 | 10.7 | 1.3 | 62.0 | 0.84 |
| Pop2 | HOH_2021 | 1.1 | 0.0 | 48.0 | 0.59 |
| Pop2 | ROS_2021 | 3.3 | 0.0 | 86.8 | 0.13 |
| **Pop3** | **Across env.** | **3.8** | **1.2** | **34.4** | **0.64** |
| Pop3 | HOH_2020 | 8.9 | 1.1 | 49.8 | 0.00 |
| Pop3 | HOH_2021 | 0.2 | 0.0 | 37.8 | 0.03 |
| Pop3 | ROS_2021 | 0.2 | 0.0 | 36.4 | 0.00 |
| **Pop4** | **Across env.** | **4.5** | **1.0** | **37.9** | **0.60** |
| Pop4 | HOH_2020 | 10.0 | 2.3 | 43.0 | 0.00 |
| Pop4 | HOH_2021 | 0.2 | 0.0 | 28.7 | 0.07 |
| Pop4 | ROS_2021 | 0.2 | 0.0 | 67.3 | 0.01 |
| **Pop5** | **Across env.** | **3.7** | **0.1** | **36.7** | **0.46** |
| Pop5 | DAL_2021 | 0.7 | 0.0 | 29.5 | 0.08 |
| Pop5 | HOH_2021 | 0.2 | 0.0 | 37.5 | 0.08 |
| Pop5 | SOL_2021 | 4.3 | 0.2 | 47.3 | 0.66 |
| **Pop6** | **Across env.** | **7.1** | **2.1** | **18.2** | **0.37** |
| Pop6 | DAL_2021 | 9.1 | 0.0 | 25.5 | 0.30 |
| Pop6 | HOH_2021 | 3.7 | 0.0 | 17.1 | 0.34 |
| Pop6 | SOL_2021 | 7.0 | 0.6 | 39.7 | 0.55 |
| **Pop7** | **Across env.** | **9.1** | **0.9** | **33.2** | **0.76** |
| Pop7 | DAL_2021 | 7.2 | 0.0 | 29.9 | 0.42 |
| Pop7 | HOH_2021 | 6.8 | 0.0 | 41.6 | 0.70 |
| Pop7 | SOL_2021 | 10.0 | 0.9 | 53.2 | 0.81 |

DAL=Berlin-Dahlem, HOH=Stuttgart-Hohenheim, LEM=Lemgo, ROS=Rosenthal-Peine, SOL=Söllingen

**Table S4** Mean, minimum and maximum of yellow rust severity (%), heritabilities (H^2^) and Repeatabilities (Rep.) across environments (env.) and at the single locations, respectively (BLUEs, backtransformed)

| **Pop** | **Env.** | **Mean** | **Minimum** | **Maximum** | **H^2^/Rep.** |
| --- | --- | --- | --- | --- | --- |
| **Pop1** | **Across env.** | **10.5** | **2.6** | **39.1** | **0.90** |
| Pop1 | DAL_2020 | 6.7 | 0.6 | 38.5 | 0.83 |
| Pop1 | DAL_2021 | 4.9 | 0.0 | 26.9 | 0.56 |
| Pop1 | HOH_2020 | 7.3 | 0.0 | 41.1 | 0.78 |
| Pop1 | LEM_2020 | 27.3 | 0.0 | 80.4 | 0.87 |
| Pop1 | LEM_2021 | 7.3 | 0.3 | 58.4 | 0.78 |
| **Pop2** | **Across env.** | **2.5** | **0.2** | **33.9** | **0.82** |
| Pop2 | HOH_2020 | 0.9 | 0.0 | 47.8 | 0.20 |
| Pop2 | HOH_2021 | 0.1 | 0.0 | 10.9 | 0.11 |
| Pop2 | ROS_2020 | 3.7 | 0.4 | 49.3 | 0.91 |
| Pop2 | ROS_2021 | 1.4 | 0.0 | 64.6 | 0.05 |
| **Pop3** | **Across env.** | **6.7** | **1.3** | **37.7** | **0.76** |
| Pop3 | HOH_2020 | 1.6 | 0.0 | 61.8 | 0.33 |
| Pop3 | HOH_2021 | 0.5 | 0.0 | 43.5 | 0.40 |
| Pop3 | ROS_2020 | 10.6 | 0.5 | 48.5 | 0.75 |
| Pop3 | ROS_2021 | 20.9 | 0.0 | 58.4 | 0.34 |
| **Pop4** | **Across env.** | **7.9** | **0.4** | **37.9** | **0.78** |
| Pop4 | HOH_2020 | 2.7 | 0.0 | 49.0 | 0.47 |
| Pop4 | HOH_2021 | 1.1 | 0.0 | 44.5 | 0.61 |
| Pop4 | ROS_2020 | 14.2 | 0.9 | 64.1 | 0.92 |
| Pop4 | ROS_2021 | 26.5 | 0.0 | 60.1 | 0.56 |
| **Pop5** | **Across env.** | **0.6** | **0.0** | **66.4** | **0.61** |
| Pop5 | DAL_2021 | 0.1 | 0.0 | 54.7 | 0.18 |
| Pop5 | HOH_2021 | 0.0 | 0.0 | 75.2 | 0.20 |
| Pop5 | SOL_2021 | 1.6 | 0.0 | 70.5 | 0.27 |
| **Pop6** | **Across env.** | **1.8** | **0.1** | **20.5** | **0.58** |
| Pop6 | DAL_2021 | 0.5 | 0.0 | 18.4 | 0.25 |
| Pop6 | HOH_2021 | 0.4 | 0.0 | 38.9 | 0.34 |
| Pop6 | SOL_2021 | 3.4 | 0.0 | 26.9 | 0.42 |
| **Pop7** | **Across env.** | **1.9** | **0.0** | **40.8** | **0.80** |
| Pop7 | DAL_2021 | 0.4 | 0.0 | 30.8 | 0.31 |
| Pop7 | HOH_2021 | 0.4 | 0.0 | 47.3 | 0.43 |
| Pop7 | SOL_2021 | 4.5 | 0.0 | 46.3 | 0.61 |

DAL=Berlin-Dahlem, HOH=Stuttgart-Hohenheim, LEM=Lemgo, ROS=Rosenthal-Peine, SOL=Söllingen

**Table S5** Basic statistics and variance estimates for the trait stem rust () analyzed by a generalized model with logit link in the first stage and thus with parameters calculated from the second stage on the logit scale

|  | | Pop1 | | Pop2 | | Pop3 | | Pop4 | | Pop5 | | Pop6 | | Pop7 | |
| --- | --- | --- | --- | --- | --- | --- | --- | --- | --- | --- | --- | --- | --- | --- | --- |
| Parents | | Axioma x Memory | | Mocca x  LG Stamm1 | | Mocca x  LG Character | | Mocca x  LG Stamm2 | | Gedser x Memory | | Spontan x Bonanza | | Edward x KWS Montana | |
| N_Env_ | | 3 | | 3 | | 3 | | 3 | | 3 | | 3 | | 3 | |
| N_G_ | | 94 | | 86 | | 91 | | 92 | | 68 | | 97 | | 72 | |
| Mean | | -3.51 | | -2.75 | | -3.23 | | -3.05 | | -3.25 | | -2.57 | | -2.31 | |
| Min | | -5.40 | | -4.38 | | -4.41 | | -4.57 | | -6.80 | | -3.86 | | -4.74 | |
| Max | | -1.23 | | 0.07 | | -0.64 | | -0.49 | | -0.54 | | -1.50 | | -0.70 | |
| LSD | | 1.01 | | 1.26 | | 1.08 | | 1.44 | | 2.23 | | 0.97 | | 1.11 | |
| Variance SE | E | 0.24 | 0.25 | 1.00 | 1.00 | 1.18 | 1.19 | 1.32 | 1.33 | 0.17 | 0.19 | 0.19 | 0.19 | 0.03 | 0.04 |
|  | G | 0.69 | 0.13 | 0.98 | 0.18 | 0.27 | 0.06 | 0.40 | 0.10 | 0.54 | 0.18 | 0.07 | 0.03 | 0.49 | 0.11 |
|  | GxE | 0.21 | 0.04 | 0.21 | 0.05 | 0.09 | 0.04 | 0.25 | 0.07 | 0.60 | 0.12 | 0.13 | 0.03 | 0.28 | 0.05 |
| Weights | Mean | 9.73 | | 21.21 | | 60.10 | | 73.07 | | 33.05 | | 0.30 | | 1.76 | |
|  | Min | 0.02 | | 0.01 | | 0.01 | | 0.01 | | 0.01 | | 0.01 | | 0.03 | |
|  | Max | 85.09 | | 423.12 | | 423.15 | | 846.24 | | 194.33 | | 2.62 | | 194.32 | |
| H^2^ | | 0.84 | | 0.83 | | 0.64 | | 0.60 | | 0.46 | | 0.37 | | 0.76 | |
| h^2^ | | 0.77 | | 0.40 | | 0.00 | | 0.00 | | 0.40 | | 0.00 | | 0.64 | |

N_Env_= number of environments that were used to calculate best linear unbiased estimators across environments; N_G_= number of genotypes considered for respective parameters; Mean, Minimum are calculated from BLUEs; LSD= least significant difference on 5 level; Variance components (Variance) with standard errors (SE); weights = the reciprocal of the variance of the environment-wise predictions were used in the second stage model and mean, minimum (Min.) and maximum (Max.) are reported (instead of residual variance that was restricted to 1), H^2^= broad-sense heritability on entry-mean basis, h^2^= heritability when fitting the genotypes as additive kinship-matrix

**Table S6** Basic statistics and variance estimates for the trait yellow rust () analyzed by a generalized model with logit link in the first stage and thus with parameters calculated from the second stage on the logit scale

|  | | Pop1 | | Pop2 | | Pop3 | | Pop4 | | Pop5 | | Pop6 | | Pop7 | |
| --- | --- | --- | --- | --- | --- | --- | --- | --- | --- | --- | --- | --- | --- | --- | --- |
| Parents | | Axioma x Memory | | Mocca x LG Stamm1 | | Mocca x LG Character | | Mocca x LG Stamm2 | | Gedser x Memory | | Spontan x Bonanza | | Edward x KWS Montana | |
| N_Env_ | | 5 | | 4 | | 4 | | 4 | | 3 | | 3 | | 3 | |
| N_G_ | | 94 | | 86 | | 91 | | 92 | | 68 | | 97 | | 72 | |
| Mean | | -2.15 | | -3.66 | | -2.63 | | -2.45 | | -5.19 | | -3.99 | | -3.96 | |
| Min | | -3.64 | | -6.17 | | -4.34 | | -5.43 | | -10.75 | | -7.16 | | -10.86 | |
| Max | | -0.45 | | -0.67 | | -0.50 | | -0.49 | | 0.68 | | -1.36 | | -0.37 | |
| LSD | | 0.60 | | 1.53 | | 1.31 | | 1.15 | | 3.83 | | 1.97 | | 2.24 | |
| Variance SE | E | 0.63 | 0.45 | 2.41 | 1.98 | 1.45 | 1.19 | 1.47 | 1.20 | 1.70 | 1.72 | 0.36 | 0.37 | 0.54 | 0.55 |
|  | G | 0.44 | 0.07 | 1.34 | 0.27 | 0.71 | 0.14 | 0.59 | 0.12 | 2.88 | 0.67 | 0.68 | 0.17 | 2.59 | 0.54 |
|  | GxE | 0.14 | 0.02 | 0.69 | 0.09 | 0.62 | 0.07 | 0.46 | 0.05 | 0.54 | 0.14 | 0.57 | 0.10 | 0.32 | 0.08 |
| Weights | Mean | 0.50 | | 39.70 | | 6.09 | | 4.02 | | 22.80 | | 8.88 | | 10.59 | |
|  | Min | 0.03 | | 0.01 | | 0.01 | | 0.01 | | 0.04 | | 0.03 | | 0.03 | |
|  | Max | 158.64 | | 434.90 | | 434.90 | | 434.90 | | 188.42 | | 188.42 | | 188.42 | |
| H^2^ | | 0.90 | | 0.82 | | 0.76 | | 0.78 | | 0.61 | | 0.58 | | 0.80 | |
| h^2^ | | 0.67 | | 0.22 | | 0.28 | | 0.14 | | 0.42 | | 0.38 | | 0.71 | |

N_Env_= number of environments that were used to calculate best linear unbiased estimators across environments; N_G_= number of genotypes considered for respective parameters; Mean, Minimum are calculated from BLUEs; LSD= least significant difference on 5 level; Variance components (Variance) with standard errors (SE); weights = the reciprocal of the variance of the environment-wise predictions were used in the second stage model and mean, minimum (Min.) and maximum (Max.) are reported (instead of residual variance that was restricted to 1), H^2^= broad-sense heritability on entry-mean basis, h^2^= heritability when fitting the genotypes as additive kinship-matrix


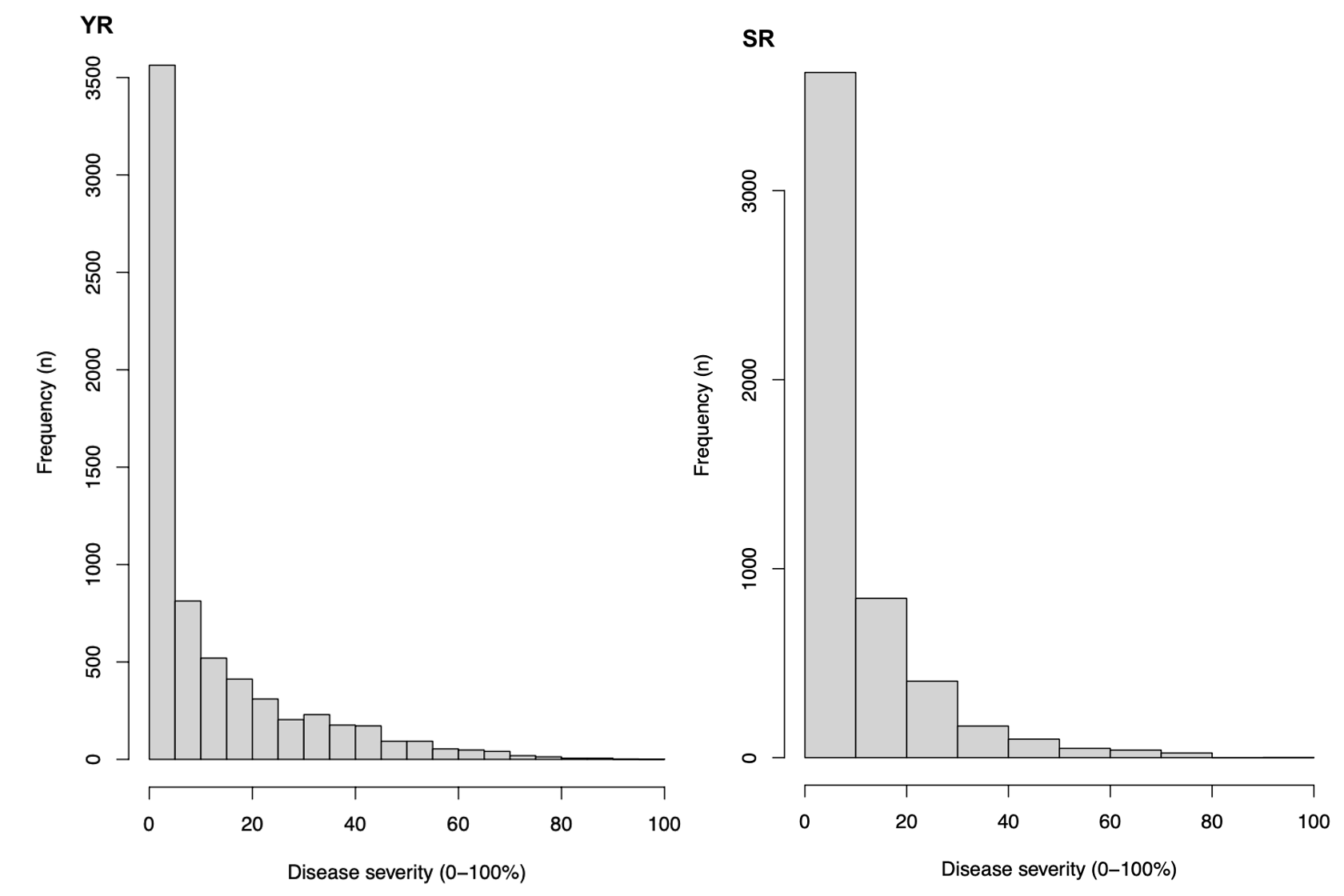


**Figure S1** Frequency distributions of the 880 winter wheat genotypes for stripe rust (YR) observations and 866 genotypes for stem rust (SR) observations from selected scoring dates across environments.
